# Feasibility of TSPO-Specific Positron Emission Tomography Radiotracer for Evaluating Paracetamol-Induced Liver Injury

**DOI:** 10.3390/diagnostics11091661

**Published:** 2021-09-10

**Authors:** Daehee Kim, Byung Seok Moon, Sun Mi Park, Sang Ju Lee, Seo Young Kang, Sanghui Park, Seung Jun Oh, Bom Sahn Kim, Hai-Jeon Yoon

**Affiliations:** 1 Department of Emergency Medicine, Incheon St. Mary’s Hospital, The Catholic University of Korea, Incheon 21431, Korea; md.kim.daehee@gmail.com; 2 Department of Nuclear Medicine, Ewha Womans University Seoul Hospital, Ewha Womans University College of Medicine, Seoul 07804, Korea; bsmoon@ewha.ac.kr (B.S.M.); psm9728@ewhain.net (S.M.P.); eironn02@gmail.com (S.Y.K.); 3 Department of Nuclear Medicine, Asan Medical Center, University of Ulsan College of Medicine, Seoul 05505, Korea; atlas425@amc.seoul.kr (S.J.L.); sjoh@amc.seoul.kr (S.J.O.); 4 Department of Pathology, Ewha Womans University Mokdong Hospital, Ewha Womans University College of Medicine, Seoul 07985, Korea; spark0430@ewha.ac.kr; 5 Department of Nuclear Medicine, Ewha Womans University Mokdong Hospital, Ewha Womans University College of Medicine, Seoul 07985, Korea

**Keywords:** paracetamol-induced liver injury, [^18^F]GE180, TSPO, positron emission tomography

## Abstract

Macrophages are activated during the early phase of paracetamol-induced liver injury (PLI). [^18^F]GE180 is a radiolabeled ligand that recognizes the macrophage translocator protein (TSPO). In this study, we evaluated the feasibility of a TSPO-specific radiotracer in a rat model of PLI. A rat model of liver injury was induced by intraperitoneal administration of paracetamol. [^18^F]GE180 positron emission tomography (PET) images were obtained after 24 h. The maximal and mean standardized uptake values (SUV*_max_* and SUV*_av_*) of the liver and serum biomarker levels were examined. The TSPO expression level was examined using real-time polymerase chain reaction and Western blot analysis. [^18^F]GE180 hepatic uptake in the PLI group was significantly higher than that in the control group (SUV*_max_ p* = 0.001; SUV*_av_ p* = 0.005). Both mRNA and protein TSPO expression levels were higher in the PLI group. The mRNA expression level of TSPO was significantly correlated with [^18^F]GE180 hepatic uptake in both groups (SUV*_max_ p* = 0.019; SUV*_av_ p* = 0.007). [^18^F]GE180 hepatic uptake in the PLI group showed a significant positive correlation with ALT_24_ and ALT_48_ (ALT_24_ *p* = 0.016; ALT_48_ *p* = 0.002). [^18^F]GE180 enabled visualization of PLI through TSPO overexpression. Our results support the potential utility of hepatic uptake by TSPO-PET as a non-invasive imaging biomarker for the early phase of PLI.

## 1. Introduction

Drug-induced liver injury (DLI) is a common adverse event encountered in clinical practice. Paracetamol, also known as acetaminophen, is one of the most widely used analgesics in Western societies and overdosing of paracetamol remains the primary cause of DLI [1,2]. A sterile inflammatory response is known to be the main mechanism of paracetamol-induced liver injury (PLI). The majority of paracetamol is metabolized by the cytochrome P450 enzyme to the reactive metabolite *N*-acetyl-p-benzoquinone imine (NAPQI), which is highly reactive, but mostly captured by glutathione (GSH). Once the GSH capturing system is saturated by paracetamol overdosing, subsequent accumulation of the reactive NAPQI triggers mitochondrial dysfunction and DNA fragmentation, resulting in hepatocyte necrosis [3]. As necrotic cell death is widespread, fulminant hepatic failure can occur, requiring liver transplantation and resulting in poor quality of life. Timely detection and therapeutic intervention at the early stages of liver injury are required to inhibit such deterioration.

Serum levels of liver-associated enzymes, such as alanine aminotransferase (ALT) and aspartate aminotransferase (AST), are currently used biomarkers of liver injury; however, they have limited value in providing a complete reflection of liver injury [4]. In particular, discriminating patients who may progress to liver failure remains a clinical challenge [5,6]. Owing to the limitations of currently available noninvasive imaging modalities, tomographic molecular imaging techniques that specifically target the discrete aspects of liver function may provide direct and quantitative visualization of cellular processes prior to irreversible deterioration [7,8].

The liver is a versatile organ with a large endogenous macrophage population, known as Kupffer cells. Activated macrophages are central effectors of sterile inflammation in the initial phase of DLI and release proinflammatory cytokines and mediators [3]. Therefore, targeting and visualization of macrophages in patients with paracetamol-overdosing may enable early detection of liver injury, as well as prediction of liver failure.

The translocator protein (TSPO), an 18 kDa protein with five transmembrane domains primarily localized in the outer membrane of mitochondria, is overexpressed in activated macrophages and can serve as an attractive surrogate marker for Alzheimer’s disease, multiple sclerosis, Huntington’s disease, various cancers, ischemic brain injury, myocarditis, DLI and rheumatoid arthritis [7,8,9,10,11,12,13,14,15,16]. From this, various TSPO-specific and -selective radiotracers have been developed to visualize by tracing microglia activation. Among them, (4S)-*N*,*N*-Diethyl-9-[2-[^18^F]fluoroethyl]-5-methoxy-2,3,4,9-tetrahydro-1*H*-carbazole-4-carboxamide ([^18^F]GE180, Flutriciclamide) is a third-generation radioligand recognizing TSPO with high affinity and specific binding [16]. The aim of this study, therefore, was to investigate the feasibility of the TSPO-specific radiotracer [^18^F]GE180 to visualize PLI using positron emission tomography (PET) by targeting activated macrophages. The relationships between [^18^F]GE180 hepatic uptake by PET imaging and ex vivo TSPO expression and serum markers of liver injury were analyzed. 

## 2. Materials and Methods

### 2.1. Synthesis of [^18^F]GE180

The TSPO-specific targeting radiotracer, [^18^F]GE180, was synthesized from the mesylate precursor ((*S*)-2-(4-(diethylcarbamoyl)-5-methoxy-3,4-dihydro-1*H*-carbazol-9(2*H*)-yl)ethyl methanesulfonate) by kryptofix-mediated nucleophilic aliphatic substitution with fluorine-18, as previously described [17] (Figures S1–S3). The isolated product with non-decay corrected radiochemical yield, calculated from trapped radioactivity on a QMA cartridge, was 36.2 ± 4.3% (*n* = 19), with over 99% of radiochemical purity. The molar activity (A_m_) was 186 ± 56 GBq/μmol in approximately 99% of radiochemical purity as determined by analytical HPLC, using UV-254 nm absorption at the end of the synthesis (Figure S4).

### 2.2. Induction of Paracetamol-Induced Liver Injury (PLI)

All animals were maintained in accordance with the National Research Council guidelines for the care and use of laboratory animals (revised in 1996). The study protocols were approved by the Institutional Animal Care and Use Committee of the Catholic University Medical College. All specific pathogen-free male Sprague Dawley rats (weight 417.9 ± 10.6 g, 13-week-old) were purchased from Orient Bio Inc. (Seongnam, Korea). Rats were acclimated in an animal room under controlled temperature (21 °C) and a 12 h light–dark cycle. The rat model of PLI was induced as described previously [18]. Twenty-five rats were subjected to fasting for 15 h, followed by intraperitoneal administration of propacetamol hydrochloride (Denogan^®^, Yungjin Pharm. Co, Ltd., Seoul, Korea) at a dose of 3 g/kg body weight to induce liver injury. Eight rats served as controls.

### 2.3. In Vivo [^18^F]GE180 PET Imaging

[^18^F]GE180 PET/CT was performed on the control (*n* = 8) and 24 h after the paracetamol injection (*n* = 21). In the induction group, four rats could not complete PET imaging because of intravenous injection failure or other issues. A dedicated small animal PET/CT scanner (NanoPET/CT, Mediso Medical Imaging Systems, Budapest, Hungary) with an 8.0 cm axial field-of-view (FOV) and a 10.0 cm transaxial FOV was used for in vivo [^18^F]GE180 PET imaging. Rats were anesthetized with 1.5% isoflurane and 24.0 ± 4.6 MBq/0.5 mL of [^18^F]GE180 was intravenously injected. PET scans were started 1 h after injection for 20 min and CT scans were used for anatomical localization of PET signals. Images were reconstructed using a three-dimensional ordered-subset expectation maximization (3D OSEM) algorithm. CT-based attenuation correction was performed.

### 2.4. PET Image Analysis

For quantitative analysis, all PET and CT images were converted into the DICOM format and analyzed using the PMOD 4.1 software (PMOD Technologies Ltd., Zurich, Switzerland). Guided by maximum intensity projection images (MIP), the SUV*_max_* and SUV*_av_* were measured by placing a 3D volume of interest (VOI) on the liver parenchyma by an experienced nuclear medicine physician. Image-derived blood [^18^F]GE180 levels were measured from the left ventricle. The SUV was calculated in pixels as (tissue radioactivity concentration)/[(injected dose)/(body weight)].

### 2.5. Serum Biochemistry Analysis

Whole blood samples were collected from the tail vein before, after 24 h and after 48 h of hepatic injury (baseline, 24 h and 48 h). The serum fraction was separated by centrifugation at 3000 rpm for 15 min. The serum levels of ALT, AST and T-Bil were measured using a fully automated spectrophotometric technique using an AU480 Chemistry Analyzer (Beckman Coulter, Inc., Fullerton, CA) using commercial kits (Chema, Italy).

### 2.6. Ex Vivo Real-Time Polymerase Chain Reaction (PCR) Analysis and Western Blot Analysis

Rats were sacrificed via CO_2_ inhalation 48 h after the hepatic injury. Real-time PCR was performed to measure the gene expression levels of TSPO and CD68 for identifying macrophages in the liver [19]. Total RNA was extracted from liver tissue using the RNeasy Plus Mini Kit (Qiagen, Hilden, Germany) and 400 ng of RNA was converted into complementary DNA using the Superscript^®^ III First-strand Synthesis System (Invitrogen Corp., Carlsbad, CA). Real-time PCR was performed using the Powerup SYBR Green master mix (ABI, Thermo Fisher Scientific, Waltham, MA) with 1 μL of complementary DNA and 0.2 μM primers on a QuantStudio 3 Real-Time PCR System (Applied Biosystems, Waltham, MA) with the following parameters: 95 °C for 2 min, 40 cycles of 95 °C for 1 s and 60 °C for 30 s. Relative expression levels were normalized to 18s ribosomal RNA expression. Primer sequences used for analysis are shown in Table S1 (see Supplementary Materials).

Western blotting was performed to measure the protein expression levels of TSPO and CD68. Total protein was isolated using radioimmunoprecipitation assay buffer (Thermo Fisher Scientific). The lysates of the samples were separated by sodium dodecyl sulfate polyacrylamide gel electrophoresis (SDS-PAGE) and transferred to polyvinylidene difluoride (PVDF) membranes (Bio-Rad Laboratories, Hercules, CA, USA). The membranes were subsequently blocked with 3% BSA for 1 h at room temperature and incubated overnight at 4 °C with primary antibodies targeting TSPO (#92291, Abcam; Cambridge, UK; diluted 1:10,000), CD68 (#31630, Abcam, diluted 1:100) and β-actin (#8227, Abcam; diluted 1:3000). Membranes were then probed with horseradish peroxidase (HRP)-conjugated anti-rabbit (#7074, Cell Signaling Technology, Danvers, MA) or anti-mouse IgG (#7076, Cell Signaling Technology). Signal intensities were measured using a ChemiDoc MP system (Bio-Rad Laboratories).

### 2.7. Immunohistochemistry

Tissue sections from rat liver were fixed with 4% paraformaldehyde for one day and then washed with water. Non-specific antibody binding was blocked with 5% normal gout serum in Tris Buffered Saline (TBS). Sections were incubated with an anti-PBR antibody (#109497, Abcam) for the TSPO receptor and anti-CD68 antibody (#31630, Abcam) for macrophage at 4 °C overnight, respectively, then with a secondary HRP-conjugated goat anti-rabbit antibody (#7074, Cell Signaling Technology) at room temperature for 1 h. The activity of HRP was detected with SignalStain^®^ Boost IHC Detection Reagent (Cell signaling Technology). Sections were counterstained using Mayer’s hematoxylin and dehydrated with ethanol, incubated with xylene and slides were mounted in Permanent mounting media (H-5000, Vector Laboratories, Burlingame, CA) with a coverslip. Sections were visualized under an inverted Olympus BX53 microscope (Olympus, Tokyo, Japan) and captured with a Q-imaging camera and ImagePro 5.1 program.

### 2.8. Statistics

All statistical analyses were performed using the MedCalc software package, version 12 (MedCalc Software, Ostend, Belgium). Independent *t*-tests were used to analyze the differences between the control and PLI groups. Paired *t*-tests were used to analyze the differences before, after 24 h and after 48 h of hepatic injury within the PLI group. Pearson’s correlation coefficient was used to evaluate the relationships between the markers. Statistical significance was set at *p* < 0.05.

## 3. Results

### 3.1. [^18^F]GE180 PET Findings as an Imaging Biomarker of Macrophage Activation in PLI

**Figure 1:**
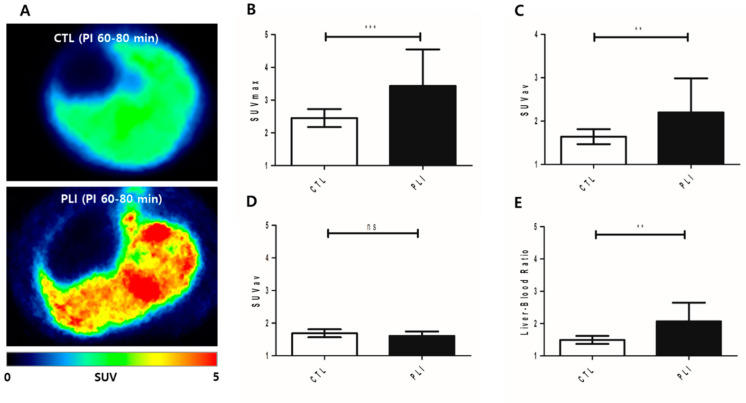
(**A**) Representative transaxial liver images acquired between 60 and 80 min post-injection (PI 60–80 min) of [^18^F]GE180 in the control (CTL, *n* = 8) and paracetamol-induced liver injury (PLI, *n* = 21) groups. (**B**) The maximal standardized uptake value (SUV*_max_*) of the liver was significantly higher in the PLI (mean ± standard deviation = 3.43 ± 1.11) than in the control group (2.45 ± 0.27). (**C**) The mean standardized uptake value (SUV*_av_*) of the liver was significantly higher in the PLI (2.20 ± 0.78) than in the control group (1.64 ± 0.17). (**D**) The SUV*_av_* of the blood did not showed significant difference between the control (mean ± SD = 1.69 ± 0.12) and PLI group (mean ± SD = 1.61 ± 0.14). (**E**) The liver-to-blood ratio was significantly higher in the PLI (mean ± SD = 2.06 ± 0.57) than in the control group (mean ± SD = 1.49 ± 0.12). Data are expressed as mean ± SD, *p < 0.05, ** *p* < 0.01, *** *p* < 0.001 by independent *t*-test.

**Figure 2:**
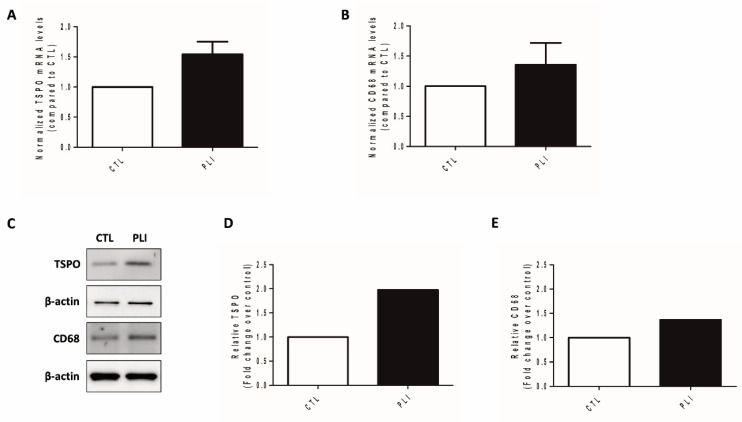
(**A**,**B**) Real-time PCR analysis results of TSPO and CD68 in the control (CTL, *n* = 8) and paracetamol-induced liver injury (PLI, *n* = 21) group. (**C**–**E**) Western blot analysis results of TSPO and CD68 in the CTL and PLI groups.

**Figure 3:**
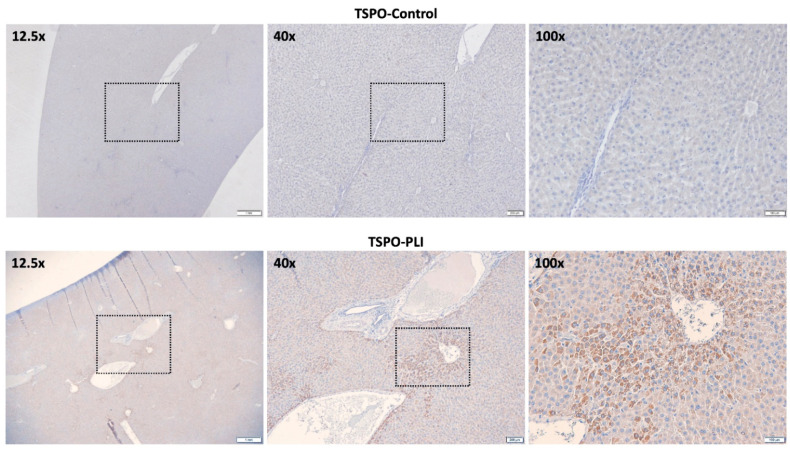
Representative light microscopic images of TSPO immunohistochemistry staining in the control and PLI group. The dotted boxes indicate the areas for the next high magnification. Scale, 1 μm for 12.5×, 100 μm for 40× and 200 μm for 100×.

**Figure 4:**
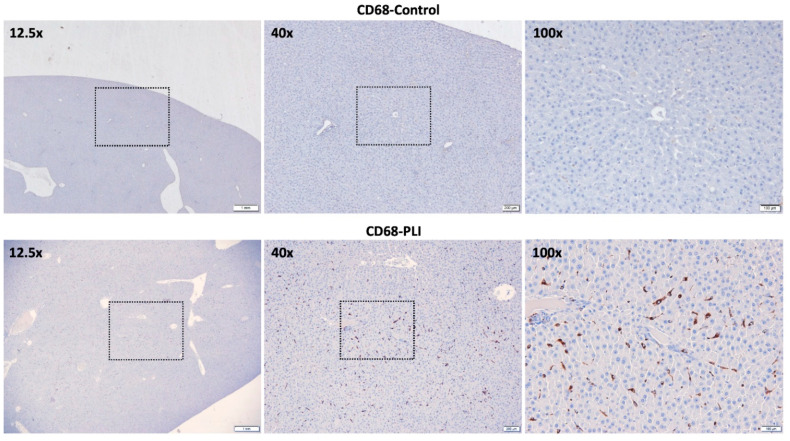
Representative light microscopic images of CD68 immunohistochemistry staining in the control and PLI group. The dotted boxes indicate the areas for the next high magnification. Scale, 1 μm for 12.5×, 100 μm for 40× and 200 μm for 100×.

On [^18^F]GE180 PET, both hepatic maximal and mean standardized uptake values (SUV*_max_* and SUV*_av_*) in the PLI group were significantly higher than those in the control group (*p* = 0.001 for SUV*_max_*, *p* = 0.005 for SUV*_av_*; Figure 1B,C). However, the blood uptake showed no significant difference between the control and PLI group (*p* = 0.117; Figure 1D). The liver-to-blood ratio in the PLI group was significantly higher than in the control group (*p* = 0.009; Figure 1E). The results of real-time polymerase chain reaction (PCR) showed an approximately 1.5-fold increase in TSPO and CD68 gene expression in the liver of PLI compared to the control group (Figure 2A,B). Western blot results showed a 2.0-fold increase in TSPO and a 1.4-fold increase in CD68 protein expression in the PLI group, compared with the control group (Figure 2C–E; Figures S5–S7). Correlation analysis results between TSPO mRNA expression and [^18^F]GE180 hepatic uptake are provided as supplementary data (*p* = 0.019 for SUV*_max_*, *p* = 0.007 for SUV*_av_*; Figure S8). No correlation was noted between the hepatic SUV*_max_* and SUV*_av_* and the gene expression level of CD68 (*p* = 0.729 for SUV*_max_*; *p* = 0.468 for SUV*_av_*). In immunohistochemistry, higher TSPO and CD68 protein expression was observed in the PLI liver than in those in the control (Figure 3 and Figure 4). Interestingly, TSPO expressions were noted not only in macrophages but also in hepatocytes of zone 3.

### 3.2. [^18^F]GE180 PET Findings as an Imaging Biomarker of Liver Injury in PLI

**Figure 5:**
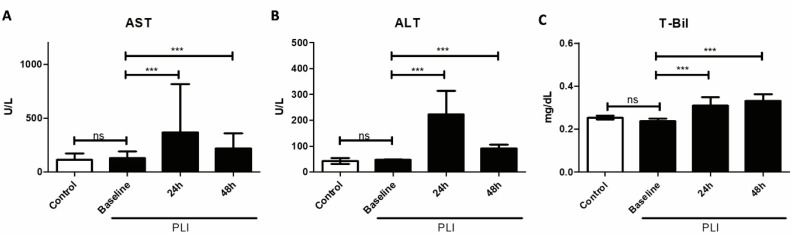
Serum levels of (**A**) alanine aminotransferase (ALT), (**B**) aspartate aminotransferase (AST) and (**C**) total bilirubin (T-Bil) were measured before, after 24 h and after 48 h of hepatic injury (baseline, 24 h and 48 h). Data are expressed as mean ± standard deviation (*n =* 8 for control and *n* = 21 for PLI), * *p* < 0.05, ** *p* < 0.01, *** *p* < 0.001, independent t-test for control vs. baseline, paired t-test for baseline vs. 24 h and baseline vs. 48 h.

Serum biomarker levels for hepatocellular injuries, such as AST and ALT, increased abruptly after 24 h of paracetamol administration (all *p* < 0.001; Figure 5A,B). After 48 h of hepatic injury, serum AST and ALT levels declined, but were still significantly higher than baseline (all *p* < 0.001). The level of total bilirubin (T-Bil) increased significantly after 24 and 48 h of paracetamol administration (Figure 5C).

**Figure 6:**
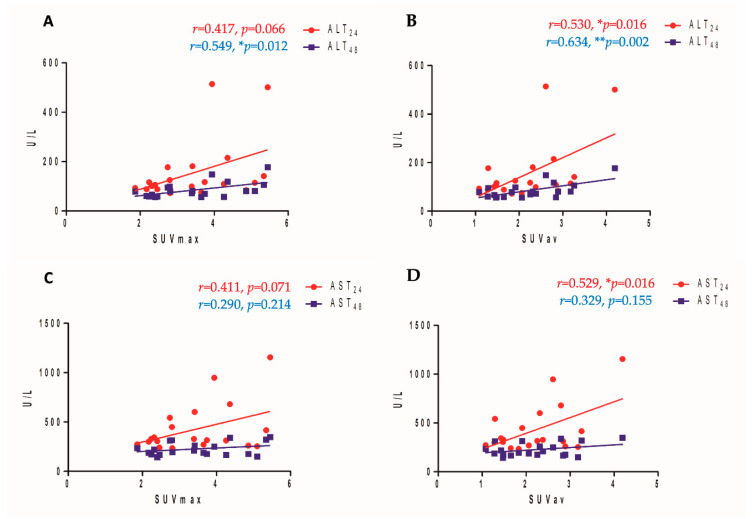
Correlation analysis results between ALT levels after 24 h and after 48 h of hepatic injury and [^18^F]GE180 hepatic uptake: (**A**) SUV*_max_*; (**B**) SUV*_av_*. Correlation analysis results between AST levels after 24 h and after 48 h of hepatic injury and [^18^F]GE180 hepatic uptake: (**C**) SUV*_max_*; (**D**) SUV*_av_*.

The hepatic SUV*_av_* in the PLI group showed a significant positive correlation with ALT_24_ and ALT_48_ (*p* = 0.016 for ALT_24_ and *p* = 0.002 for ALT_48_). The hepatic SUV*_max_* in the PLI group showed a significant positive correlation with ALT_48_ (*p* = 0.066 for ALT_24_ and *p* = 0.012 for ALT_48_). For AST, the hepatic SUV*_av_* in the PLI group showed a significant positive correlation with AST_24_ (*p* = 0.016). However, no significant correlations were noted between hepatic SUV*_av_* and AST_48_ (*p*=0.155), hepatic SUV*_max_* and ALT_24_ (*p* = 0.071) and ALT_48_. (*p* = 0.214). The hepatic SUV*_max_* and SUV*_av_* showed no significant correlations with either T-Bil_24_ or T-Bil_48_ (all *p* > 0.05). All scatter plots are shown in Figure 6.

## 4. Discussion

In this study, PET noninvasively visualized liver injury using the TSPO-specific radiotracer [^18^F]GE180. After the induction of PLI, a significant increase in the in vivo [^18^F]GE180 hepatic uptake corresponded to ex vivo TSPO expression. The increase in the in vivo [^18^F]GE180 hepatic uptake correlated significantly with an increase in liver enzymes, which is the current standard of monitoring liver injury.

PLI is estimated to account for approximately 50% of acute liver failure and approximately 30,000 patients are admitted to hospitals every year in the United States [20]. Paracetamol overdose can occur with or without awareness and there is no known difference in susceptibility to acute liver failure and liver transplantation between the two groups [21]. The most important determinant of paracetamol-induced hepatotoxicity is the ingested dose of analgesics, but, in most cases, medical staff estimate the dose based on the history provided by the patients or guardians; thus, it is difficult to accurately determine the actual dose taken.

Biochemical approaches have been used to monitor liver injury in patients with paracetamol overdose. However, serum markers can be diluted across the entire blood volume and confounded during the course of PLI in cases where various processes occur simultaneously [22]. Moreover, predicting subjects that may progress to liver failure remains a clinical challenge [5,6]. Liver biopsy is considered the gold standard for identifying drug-induced hepatotoxicity, but this invasive procedure is not routinely used in clinical practice [23]. Since the volume of tissue obtained from biopsy covers only a small part of the entire liver, the sampling error is inevitably high when the disease manifests heterogeneously in PLI, despite its invasiveness [24]. Therefore, noninvasive PET imaging has the advantage of providing a view of the entire liver in vivo and eliminating sampling errors. In this study, we applied activated macrophage-targeted TSPO PET imaging for the first time to evaluate PLI.

The role of macrophages in the pathogenesis of PLI remains controversial. Activated macrophages mediate the production of proinflammatory cytokines, thus contributing to paracetamol-induced hepatotoxicity [25]. However, other studies have suggested the protective role of hepatic macrophages by reducing inflammation and promoting hepatic regeneration [26]. Michael et al. reported that the resolution of hepatic damage from paracetamol-induced hepatotoxicity was delayed by the depletion of paracetamol-induced macrophages [27]. Cynthia et al. also reported the protective role of Kupffer cells by identifying a significant decrease in the mRNA expression levels of interleukin (IL)-6, IL-10 and IL-18 hepato-regulatory cytokines after Kupffer cell depletion [26]. On the other hand, Laskin et al. found blockage of hepatic tissue injury by the inhibition of hepatic macrophages and suggested that macrophages directly contribute to PLI [28]. Mossanen et al. demonstrated massive recruitment of monocytes into the paracetamol-poisoned liver. They suggested that monocyte-derived macrophages (MoMFs) exacerbate inflammation and injury during the early phase of PLI, although MoMFs can express both proinflammatory and tissue-repair genetic profiles [29]. This controversy may be due to the plasticity of macrophages and the difficulty in distinguishing subpopulations of macrophages. A recent study by Tsuji et al. analyzed paracetamol-induced hepatotoxicity according to M1/M2-macrophage subtypes and reported that proinflammatory M1-macrophages increased significantly on days 1 and 2, whereas tissue-repairing M2-macrophages appeared later, on days 2 and 3 [18]. The imbalance between the expression of the two macrophage subtypes may contribute to the progression of PLI.

In addition, identifying macrophage phenotypes is important, in that TSPO expression can be differ by phenotype. In vitro studies on murine macrophages reported a significant increase in TSPO expression after M1 activation, or no significant phenotype-dependent differences [15,30]. Meanwhile, Narayan et al. investigated TSPO expression in human macrophages and found a significant difference in TSPO expression between M1 and M2 phenotypes [31]. In this study, we showed that the expression of CD68, which is widely used as a pan-macrophage marker, is increased in the liver of the PLI group. Further study is required at the phenotype level.

Immunohistochemistry revealed higher TSPO and CD68 protein expressions in the PLI liver than those in the control (Figure 3 and Figure 4). Interestingly, TSPO expressions were observed in hepatocytes of zone 3, as well as macrophages. Necrosis of hepatocytes starting in zone 3 is a histopathological indicator of PLI. TSPO is ubiquitously expressed and also found at low levels in hepatocytes but is elevated upon liver injuries [32]. Though TSPO is mainly found in macrophages, considering hepatocytes occupy more than 70% of the liver, the increase in TSPO expression in non-macrophages (e.g., hepatocytes) suggests the potential of [^18^F]GE180 PET as an imaging technique that can more sensitively reflect paracetamol-induced liver injury. In addition, the larger gap between the control and PLI in TSPO than CD68 from the PCR and Western blot analysis is interpreted to be due to TSPO expression in injured hepatocytes. Further studies are needed on the role of TSPO expression in hepatocytes in paracetamol-induced hepatotoxicity.

In this study, we found an increase in [^18^F]GE180 hepatic uptake after 24 h of paracetamol-overdosing and the increase was correlated significantly with the increase in AST and ALT levels. Between AST and ALT, [^18^F]GE180 hepatic uptake showed better correlations with ALT than AST. Because elevated AST levels indicate nonspecific tissue injury rather than liver-specific tissue damage, AST levels can be affected by stressful procedures (e.g., blood sampling), whereas any increase in ALT level is a direct indicator of liver injury.

Studies regarding the development of non-invasive molecular imaging techniques for PLI evaluation have been conducted by several researchers. However, most of the studies are preclinical studies using fluorescence probes targeting DLI-relevant enzymes; therefore, their clinical application is limited [33]. Unlike fluorescence imaging, PET can be applied clinically, but only a small number of studies exist. Recently, Salas et al. developed a 2-deoxy-2-[^18^F]fluoroarabinose ([^18^F]DFA) PET radiotracer to measure ribose salvage activity, which is the most active pathway in the liver [8]. They identified a decrease in hepatic [^18^F]DFA uptake after paracetamol overdose. In addition, they suggested that hepatic [^18^F]DFA uptake could distinguish between patients with PLI who were about to die and those who would survive. Discriminating between patients who may progress to liver failure is a clinical issue of great interest. Future investigations evaluating the prognostic value of [^18^F]GE180 hepatic uptake are promising.

The TSPO polymorphism is one of the major concerns hindering its rapid clinical translation. Since the development of the next-generation TSPO ligand due to the high signal-to-noise ratio of the first-generation TSPO ligand [^11^C]PK11195, the variable binding affinity caused by a single-nucleotide polymorphism of the TSPO gene has been raised as another problem for the second-generation TSPO ligand. Therefore, efforts have been made to develop third-generation TSPO ligands that are insensitive to polymorphisms. Feeny et al. investigated the radiotracer characteristics of [^18^F]GE180 in the brains of healthy human subjects and found no genotype-related effects on [^18^F]GE180 high-affinity and mixed-affinity binders [34]. Meanwhile, Fan et al. suggested two-fold higher specific binding for high-affinity binders than for mixed-affinity binders [16]. Differences in allelic status can be masked by the large effects of inflammatory activity on TSPO expression. However, the correlations between [^18^F]GE180 uptake of the target organ (in this study, liver) and histological quantification of TSPO expression in genotyped individuals should be elucidated for [^18^F]GE180 application in future clinical studies.

Furthermore, there are main limitations to our study. First, we did not perform metabolite analysis. Since [^18^F]GE180 is metabolized and about 21% of the parent present at 60 min after injection [35], the possibility that some metabolites may also contribute to the hepatic uptake on [^18^F]GE180 PET cannot be excluded. Second, the use of static protocol has to be verified through full dynamic acquisition of [^18^F]GE180 PET, because the pharmacokinetics might be different between control and PLI group. However, the time activity curves of [^18^F]GE180 in the liver of normal rat has already been investigated [36]. According to the study, the time activity curve of liver changes rapidly up to 30 min after injection and remains stable thereafter. Therefore, to simply evaluate the uptake difference between control and disease group, a static acquisition for a certain period of time of at least 30 min after injection might be sufficient. Third, a direct linear relationship between the expression level of the TSPO protein and [^18^F]GE180 hepatic uptake was not identified in this study. Although we found a trend of linear correlation between [^18^F]GE180 hepatic uptake and TSPO expression at the mRNA level, this should be verified at the protein level due to insufficient correlations between mRNA and protein expression levels.

## 5. Conclusions

[^18^F]GE180 hepatic uptake correlated well with TSPO overexpression and serum markers for liver injury, enabling visualization of PLI. The present findings support the potential utility of [^18^F]GE180-PET, which appears to be a promising tool for detecting the early stages of PLI.

## Data Availability

The data presented in this study are available from the corresponding author upon reasonable request.

## Supplementary Materials

The following are available online at https://www.mdpi.com/article/10.3390/diagnostics11091661/s1, Figure S1: Preparative HPLC separation chromatogram of reaction mixture, Figure S2: Analytical HPLC chromatogram of formulated final solution, Figure S3: Analytical HPLC chromatogram of co-injection with non-radioactive standard compound, Figure S4: Standard curve obtained from six concentrations of non-radioactive standard compound, Figure S5: The full blot results of TSPO in Western blot analysis (repeated three times in the control and PLI group), Figure S6: The full blot results of CD68 in Western blot analysis (repeated three times in the control and PLI group), Figure S7: The full blot results of β-actin in Western blot analysis (repeated three times in the control and PLI group), Figure S8: Correlation analysis results between TSPO mRNA expression and [^18^F]GE180 hepatic uptake (A: SUV*_max_*, B: SUV*_av_*), Table S1: Primer sequences used for real-time, reverse-transcriptase PCR.
