# Supplementary material for: Feasibility of TSPO-Specific Positron Emission Tomography Radiotracer for Evaluating Paracetamol-Induced Liver Injury
Source: Diagnostics (Basel). 2021 Sep 10;11(9):1661. doi: 10.3390/diagnostics11091661 (PMC8467059; doi:10.3390/diagnostics11091661)
Supplement: Supplementary file 1 [file diagnostics-11-01661-s001.zip › diagnostics-1354361-supplementary.pdf]

Supplementary Materials

Feasibility of TSPO-specific positron emission tomography radiotracer for evaluating paracetamol-induced liver injury

Daehee Kim [1,†], Byung Seok Moon [2,†], Sun Mi Park [2], Sang Ju Lee [3], Seo Young Kang [2], Sanghui Park [4], Seung Jun Oh [3], Bom Sahn Kim [2,*] and Hai-Jeon Yoon [5,*]

[1] *Department of Emergency Medicine, Incheon St. Mary's Hospital, The Catholic University of Korea, Incheon 21431, Korea; md.kim.daehee@gmail.com (D.K.)*
[2] *Department of Nuclear Medicine, Ewha Womans University Seoul Hospital, Ewha Womans University College of Medicine, Seoul 07804, Korea; bsmoon@ewha.ac.kr (B.S.M.); psm9728@ewhain.net (S.M.P.); eironn02@gmail.com (S.Y.K.)*
[3] *Department of Nuclear Medicine, Asan Medical Center, University of Ulsan College of Medicine, Seoul 05505, Korea; atlas425@amc.seoul.kr (S.J.L.); sjoh@amc.seoul.kr (S.J.O.)*
[4] *Department of Pathology, Ewha Womans University Mokdong Hospital, Ewha Womans University College of Medicine, Seoul 07985, Korea; spark0430@ewha.ac.kr (S.P.)*
[5] *Department of Nuclear Medicine, Ewha Womans University Mokdong Hospital, Ewha Womans University College of Medicine, Seoul 07985, Korea*

* Correspondence: kbomsahn@ewha.ac.kr (B.S.K.); haijeon.yoon@gmail.com (H.-J.Y.)
† These authors contributed equally to this work.

Radiochemistry.

Figure S1. Preparative HPLC separation chromatogram of reaction mixture.

Figure S2. Analytical HPLC chromatogram of formulated final solution.

Figure S3. Analytical HPLC chromatogram of co-injection with non-radioactive standard compound.

Figure S4. Standard curve obtained from six concentrations of non-radioactive standard compound.

Figure S5. Full blot results of TSPO in Western blot analysis (repeated three times in the control and PLI group).

Figure S6. Full blot results of CD68 in Western blot analysis (repeated three times in the control and PLI group).

Figure S7. Full blot results of β-actin in Western blot analysis (repeated three times in the control and PLI group).

Figure S8. Correlation analysis results between TSPO mRNA expression and [^{18}F]GE180 hepatic uptake (A: SUV_{max}, B: SUV_{av}).

Table S1. Primer sequences used for real-time, reverse-transcriptase PCR

Radiochemistry. All commercial reagents and solvents were used without further purification unless otherwise specified. Reagents and solvents were commercially purchased from Sigma-Aldrich (U.S.A.). Radio-TLC were performed on Merck silica gel glass plates (60 F_{254}) and analyzed on a Bioscan radio-TLC scanner (USA). All radioactivities were measured using a VDC-606 activity calibrator from Veenstra Instruments (Netherlands). $H_2{}^{18}O$ was purchased from Wo Isotope Co., Ltd. (China). ^{18}F-Fluoride was produced at Ewha Womans University Seoul Hospital by $^{18}O(p,n)^{18}F$ reaction through proton irradiation using a GE PETtrace cyclotron (GE Healthcare, U.S.A.). QMA carbonate plus light and C18 plus Sep-Pak® cartridges were purchased from Waters Corp. (USA). HPLC purification was performed with a Gilson 322 system (Waters, Xterra shield RP18 OBD preparative column, 10 μm, 10 x 250 mm) equipped with a UV detector (Wavelength set at 254 nm) and a gamma-ray detector (LabLogic, USA). A mixture of acetonitrile and water (50:50) was used as the mobile phase at a flow rate of 3 mL/min. The purified radiotracer and non-radioactive authentic compound were analyzed in acetonitrile and water (55:45) as the mobile phase at a flow rate of 1 mL/min using Agilent 1260 infinity system (USA). The HPLC system (Waters, Xterra RP18, 4.6 × 250 mm, 5 μm) was equipped with a NaI radiodetector (Raytest, Germany) and a UV-detector (254 nm). HPLC-grade solvents (J. T. Baker, USA) were used for HPLC purification after membrane filtration (Whatman, 0.22 μm).

The TSPO-specific targeting radiotracer, (4S)-*N*,*N*-Diethyl-9-[2-^{18}F-fluoroethyl]-5-methoxy-2,3,4,9-tetrahydro-1*H*-carbazole-4-carboxamide (^{18}F-GE180, Flutriciclamide) was synthesized from the mesylate precursor, (*S*)-2-(4-(diethylcarbamoyl)-5-methoxy-3,4-dihydro-1*H*-carbazol-9(2*H*)-yl)ethyl methanesulfonate, by kryptofix-mediated nucleophilic aliphatic ^{18}F-substitution with a slight modification according to the procedures described in the literature [Wadsworth H. et al. *Bioorg Med Chem Lett* **2012**, *22*, 1308; doi:10.1016/j.bmcl.2011.12.084]. Briefly, [^{18}F]fluoride was supplied from cyclotron. Received F-18 (3.7-5.5 GBq) in O-18 water was extracted on the QMA carbonate (preactivated with 10 mL of water) by Kryptofix 2.2.2 ($K_{2.2.2}$, 5.0 mg) and $KHCO_3$ (1.0 mg) in MeOH:H_2O mixture (1 mL, 9:1, v/v). The mixture was dried azeotropically by nitrogen streaming at 100 °C and repeated with subsequent addition of CH_3CN (0.4 mL). After drying, the mesylate precursor (1.0-1.2 mg) in acetonitrile (0.5 mL) was added to the reaction vessel and heated at 100 °C for 10 min. The reaction mixture was cooled to room temperature and diluted with 10 mL of water, loaded on the C18 plus Sep-Pak cartridge, washed with 10 mL of water and then eluted with 1.5 mL of CH_3CN. The eluted CH_3CN solution was diluted with 1.5 mL of water, filtered by HPLC filter (UHP, 0.45 μm) and purified with reverse-phase HPLC system. The desire product fraction was collected around 16.5 min of retention time (Figure S1) and diluted with 20 mL of water. The solution was exchanged to approximate 8% EtOH/saline solution by a C18 plus Sep-Pak cartridge to remove the biologically unavailable HPLC solvent. The radiochemical

purity was checked using analytical HPLC system (Xterra RP18, 4.6 × 250 mm, 5 μm) (Figure S2) and identity was confirmed by co-injection with non-radioactive authentic compound (Figure S3). The isolated product with non-decay corrected radiochemical yield, calculated from trapped radioactivity on a QMA cartridge, was 36.2 ± 4.3% (n = 19) with an over 99% of radiochemical purity. The molar activity (A_m) was 186 ± 56 GBq/μmol in approximately 99% of radiochemical purity as determined by analytical HPLC, using UV-254 nm absorption at the end of the synthesis (Figure S4). It was determined by integration of UV area after 20 μL of final formulated solution was reinjected to analytical HPLC system and calculated from activity/mol by standard curve ($y = 258,573.8x + 0.89$, $r^2 = 0.9999$, Figure S4), previously obtained with six concentrations of standard compound (1.444, 0.722, 0.361, 0.144, 0.072 and 0.014 nmol).

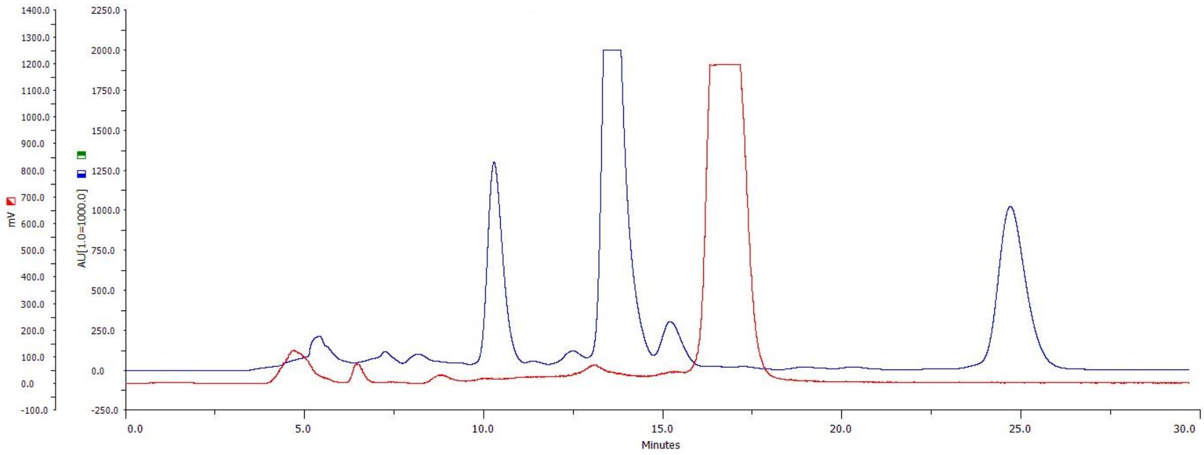

Figure S1. Preparative HPLC separation chromatogram of reaction mixture (Xterra shield RP18 OBD preparative column, 10 μm, 10 x 250 mm; UV-254 nm (blue line) and a gamma-ray (red line); eluent: 50% $CH_3CN:H_2O$; flow rate: 3 mL/min).

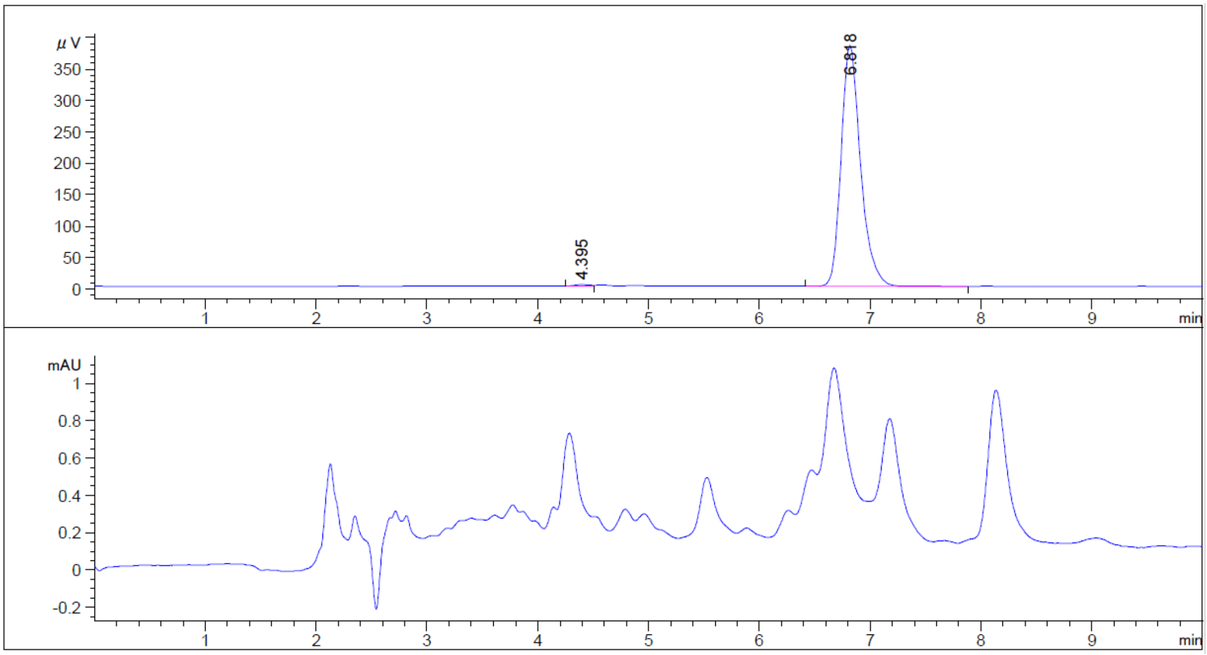

Figure S2. Analytical HPLC chromatogram of formulated final solution (Xterra RP-18 column, 5 µm, 4.6 × 250 mm; UV-254 nm (bottom) and a gamma-ray (upper); eluent: 55% CH_3CN:H_2O; flow rate: 1 mL/min).

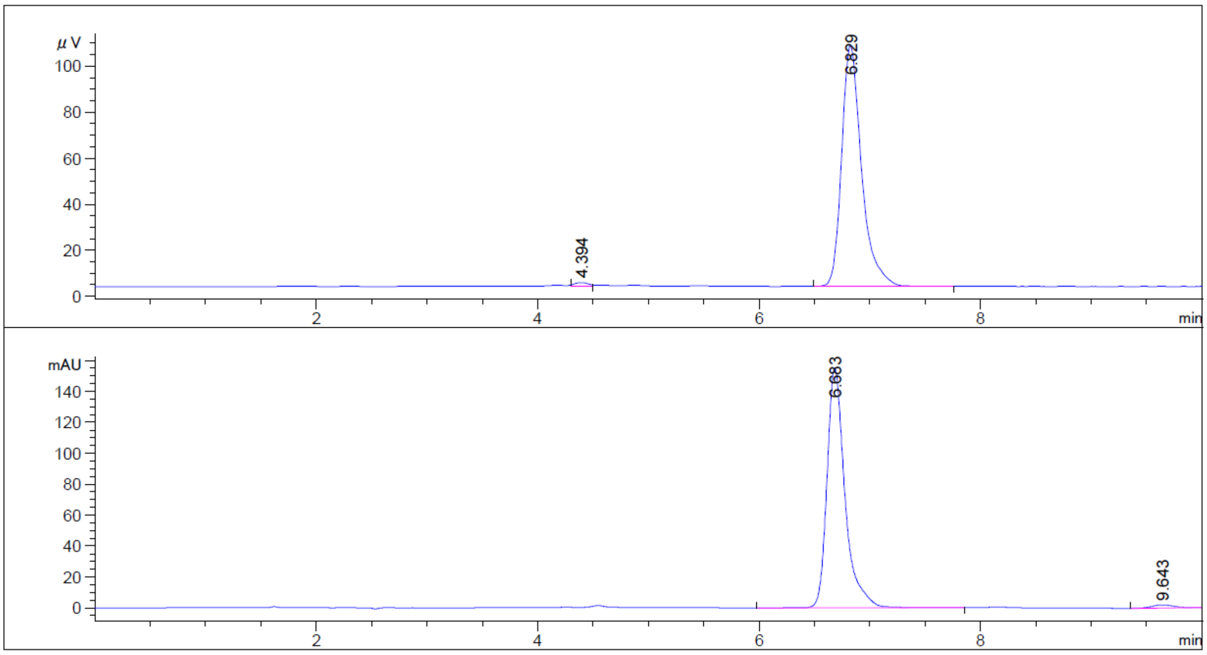

Figure S3. Analytical HPLC chromatogram of co-injection with non-radioactive standard compound (Xterra RP-18 column, 5 µm, 4.6 × 250 mm; UV-254 nm (bottom) and a gamma-ray (upper); eluent: 55% CH_3CN:H_2O; flow rate: 1 mL/min).

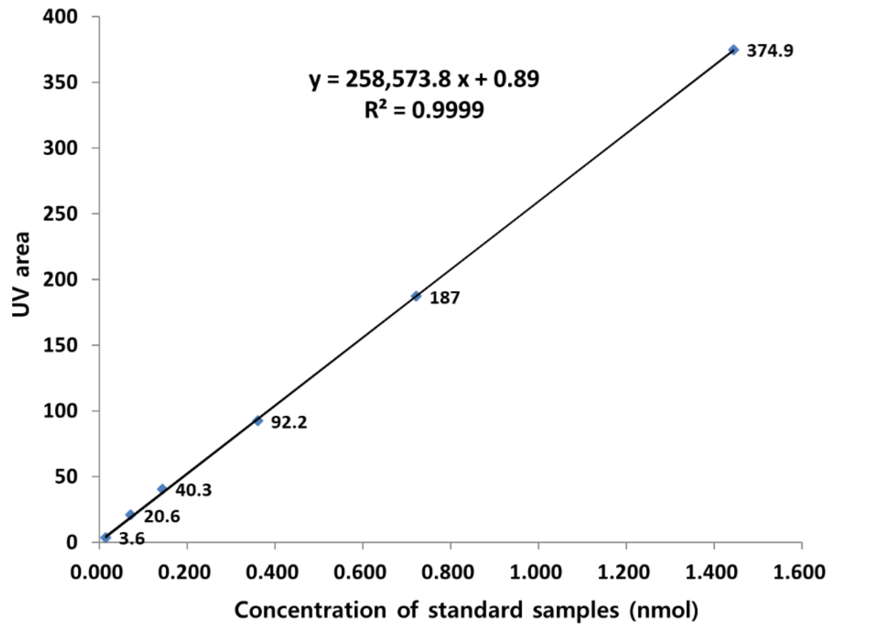

Figure S4. Standard curve obtained from six concentrations of non-radioactive standard compound (1.444, 0.722, 0.361, 0.144, 0.072 and 0.014 nmol).

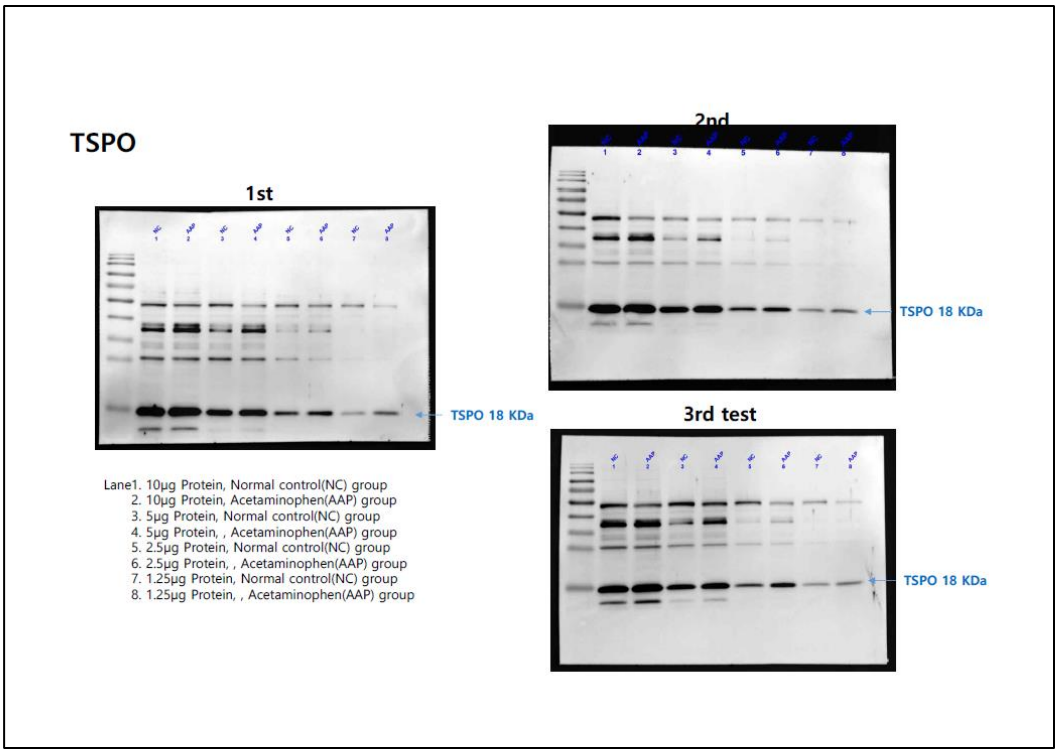

Figure S5. Full blot results of TSPO in Western blot analysis (repeated three times in the control and PLI group).

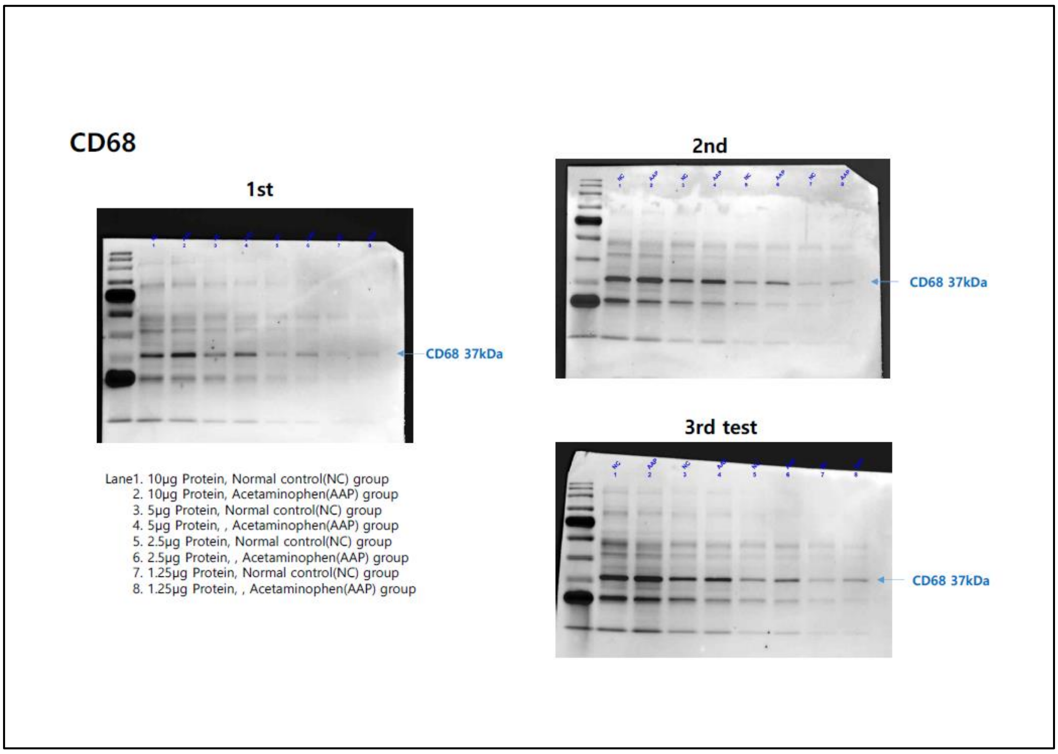

Figure S6. Full blot results of CD68 in Western blot analysis (repeated three times in the control and PLI group).

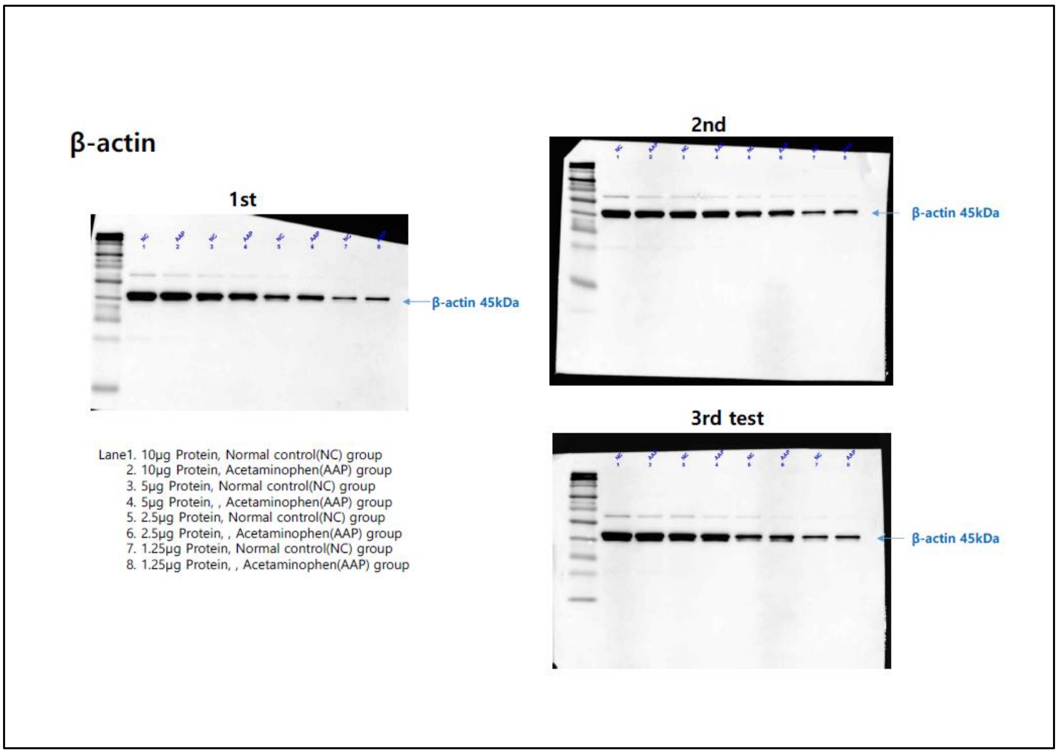

Figure S7. Full blot results of β-actin in Western blot analysis (repeated three times in the control and PLI group).

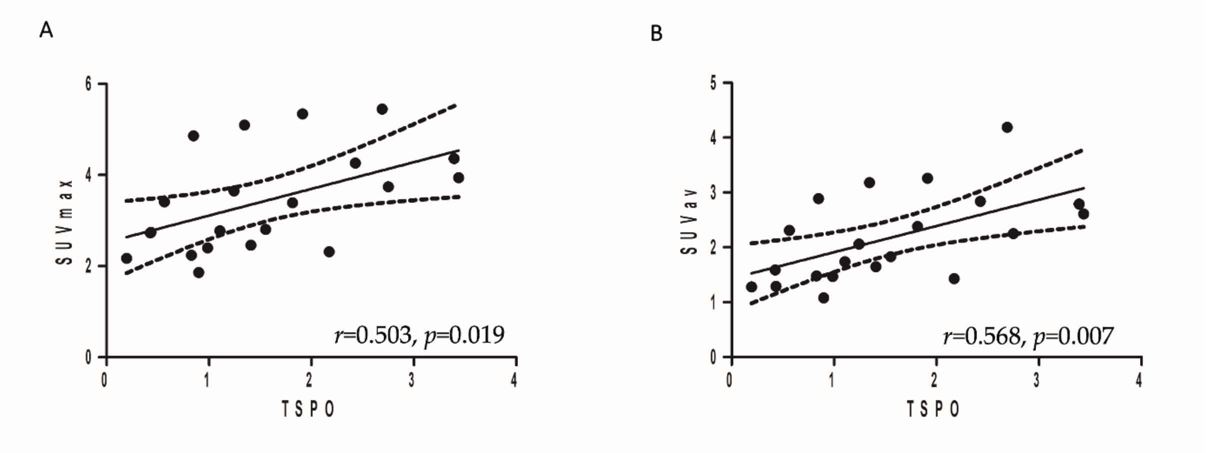

Figure S8. Correlation analysis results between TSPO mRNA expression and [^{18}F]GE180 hepatic uptake (A: SUV$_{max}$, B: SUV$_{av}$).

Table S1. Primer sequences used for real-time, reverse-transcriptase PCR

Genes	Forward	Reverse	Size (bp)
TSPO	AGAAACCCTCTTGGCATCCG	CGTCCTCTGTGAAACCTCCC	120
CD68	CAAAAAGGCTGCCACTCTTC	GTGGGAGAAACTGTGGCATT	231
18s rRNA	CGCTACACTGAACTGGCTCA	TGTGTACAAAGGGCAGGGAC	174
